# Adherence to Antiretroviral Therapy and Acceptability of Planned Treatment Interruptions in HIV-Infected Children

**DOI:** 10.1007/s10461-012-0197-y

**Published:** 2012-05-15

**Authors:** Linda Harrison, Jintanat Ananworanich, Djamel Hamadache, Alexandra Compagnucci, Martina Penazzato, Torsak Bunupuradah, Antonio Mazza, Jose Tomas Ramos, Jacquie Flynn, Osvalda Rampon, Maria Jose Mellado Pena, Daniel Floret, Magdalena Marczynska, Ana Puga, Silvia Forcat, Yoann Riault, Marc Lallemant, Hannah Castro, Diana M. Gibb, Carlo Giaquinto, On Behalf of the Paediatric European Network for Treatment of AIDS (PENTA) 11 Trial Team

**Affiliations:** 1 Medical Research Council Clinical Trials Unit, Aviation House, 125 Kingsway, London, WC2B 6NH UK; 2 HIV-NAT, The Thai Red Cross AIDS Research Centre, Bangkok, Thailand; 3 Imperial College Healthcare NHS Trust, London, UK; 4 INSERM SC10, Paris, France; 5 Department of Paediatrics, University of Padova, Padua, Italy; 6 Ospedale S. Chiara, Trento, Italy; 7 Hospital Universitario de Getafe, Madrid, Spain; 8 Great Ormond Street Hospital for Children NHS Trust, London, UK; 9 Instituto de Salud Carlos III, Madrid, Spain; 10 Hôpital Femme-Mère-Enfant, Lyon, France; 11 Medical University of Warsaw, Warsaw, Poland; 12 Childrens Diagnostic and Treatment Center, Fort Lauderdale, USA; 13 Institut de Recherche pour le Développement (IRD), Chiang Mai University, Chiang Mai, Thailand

**Keywords:** Adherence, Acceptability, Planned treatment interruptions, HIV, Children

## Abstract

There have been no paediatric randomised trials describing the effect of planned treatment interruptions (PTIs) of antiretroviral therapy (ART) on adherence, or evaluating acceptability of such a strategy. In PENTA 11, HIV-infected children were randomised to CD4-guided PTIs (*n* = 53) or continuous therapy (CT, *n* = 56). Carers, and children if appropriate, completed questionnaires on adherence to ART and acceptability of PTIs. There was no difference in reported adherence on ART between CT and PTI groups; non-adherence (reporting missed doses over the last 3 days or marking <100 % adherence since the last clinical visit on a visual analogue scale) was 18 % (20/111) and 14 % (12/83) on carer questionnaires in the CT and PTI groups respectively (odds ratios, OR (95 % CI) = 1.04 (0.20, 5.41), χ^2^ (1) = 0.003, *p* = 0.96). Carers in Europe/USA reported non-adherence more often (31/121, 26 %) than in Thailand (1/73, 1 %; OR (95 % CI) = 54.65 (3.68, 810.55), χ^2^ (1) = 8.45, *p* = 0.004). The majority of families indicated they were happy to have further PTIs (carer: 23/36, 64 %; children: 8/13, 62 %), however many reported more clinic visits during PTI were a problem (carer: 15/36, 42 %; children: 6/12, 50 %).

## Resumen

No ha habido estudios pediátricos randomizados que describan el efecto de las interrupciones planificadas del tratamiento (IPT) antirretroviral en el seguimiento del tratamiento o en la aceptabilidad de dicha estrategia. En PENTA11, niños infectados con HIV fueron randomizados o bien en IPT guiadas por sus CD4 (*n* = 53) o bien en terapia continua (TC, *n* = 56). Tanto los cuidadores como los niños, cuando era apropiado, completaron cuestionarios de seguimiento de la terapia antirretroviral y de aceptabilidad de las interrupciones. No se encontró diferencia en cuanto al seguimiento del tratamiento entre los dos grupos. No seguimiento (dosis perdidas durante los últimos 3 días o <100 % desde la ultima visita a la clínica en una escala visual analógica) fue del 18 % (20/111) y del 14 % (12/183) en los cuestionarios de los cuidadores en TC y en IPT respectivamente (odds ratios, OR (95 % CI) = 1.04 (0.20, 5.41), χ^2^ (1) = 0.003, *p* = 0.96). Los cuidadores en Europa/USA informaron de un no seguimiento del tratamiento más a menudo (31/121, 26 %) que en Tailandia (1/73, 1%; OR (95 % CI) = 54.65 (3.68, 810.55), χ^2^ (1) = 8.45, *p* = 0.004). La mayoría de las familias indicaron que les gustaría realizar más ITP (cuidador: 23/36, 64 %; niños: 8/13, 62 %), sin embargo muchos indicaron que el mayor numero de visitas a la clínica durante la IPT era un problema (cuidador: 15/35, 43 %; niños: 6/12, 50 %).

## Introduction

AIDS related mortality and morbidity has declined substantially in HIV-infected children since the introduction of combination antiretroviral therapy (ART) [1, 2]. However, complete HIV suppression requires a high level of adherence to ART to be sustained over a lifetime [3, 4]. In children, the life-long exposure to treatment also raises concerns regarding potential long-term toxicity such as lipodystrophy, osteopenia, and mitochondrial dysfunction [5, 6]. Additionally, treatment sequencing, given the greater potential for inadequate dosing and the absence of appropriate licensed drug formulations, is often challenging [7]. Therefore, planned treatment interruptions (PTIs) may be welcomed by children and their families, but may also have a negative impact on adherence once ART is re-started.

Trials evaluating treatment interruptions in adults have reported higher rates of AIDS events/death and serious non-AIDS events in those stopping ART [8–12], and the SMART trial reported that CD4-guided episodic use of ART resulted in inferior quality of life (QOL) [13]. PENTA 11 was a pilot study evaluating CD4-guided PTIs in HIV-infected children and the key finding of the trial was that no serious clinical outcomes were reported in children undergoing PTIs [14]. Nevertheless, there was a significant excess of minor clinical events in the PTI group after stopping ART, compared to children on continuous therapy (CT), which may have been problematic for children and families.

There have been no paediatric studies describing the effect of PTIs on adherence within a randomised trial, or evaluating acceptability of such a strategy. In the PENTA 11 trial, adherence to ART and acceptability of PTIs was measured routinely, and this paper presents findings from analyses of these data.

## Methods

### Trial Design

**Fig. 1:**
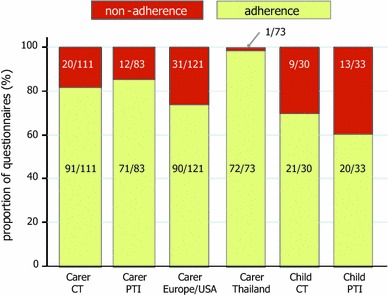
Non-adherence during follow-up by randomised group and region. Non-adherence was defined as reporting one or more missed doses over the last 3 days and/or marking <100 % adherence since the last clinical visit on the VAS. All child questionnaires were completed in Europe/USA. *CT* continuous therapy, *PTI* planned treatment interruption

PENTA 11 was an open, multicentre, randomised, phase II, trial (ISRCTN36694210) in HIV-infected children aged 2–15 years, on any ART regimen containing ≥3 drugs which had been taken for ≥24 weeks. Eligibility to participate also required that the two most recent plasma HIV-1 RNA measurements were <50 copies/ml and the two most recent CD4 % measurements were ≥30 % (ages 2–6 years) or ≥25 % and CD4 count ≥500 cells/mm^3^ (7–15 years) [14]. Children were randomised in a 1:1 ratio to either continue ART (CT) or stop ART and then follow a strategy of CD4-guided PTI (ART was restarted if confirmed CD4 % was less than 20 % or more than 48 weeks had been spent off ART; ART could be stopped again after 24 weeks on ART and confirmed CD4 % was ≥30 % (ages 2–6 years) or ≥25 % and CD4 count ≥500 cells/mm^3^ (7–15 years); see Fig. 1 in the main trial publication for further details on the design of the trial [14]), for at least 72 weeks. The protocol was approved by the ethics committee for each participating centre (listed in Acknowledgments). All parents/guardians gave written consent, and children gave written assent, according to their age and knowledge of HIV status.

### Adherence Questionnaires

Carers, and children if appropriate, were asked to complete adherence questionnaires, adapted from previous PENTA studies [15, 16], at baseline, weeks 24, 48 and 72 in the CT group, and at baseline, then at 4, 12, 24 and 48 weeks after each ART re-start in the PTI group. Questionnaires were completed at the time of scheduled clinic visits; with the help of the nurse or doctor if required.

Adherence was assessed by a question used in previous PENTA studies to measure adherence [15, 16]; “*Can you say how many times your child has*/*you have missed a dose of antiretroviral medicines over the last 3* *days*?” Additionally, carers, and children if appropriate, were asked to mark adherence to ART since the last clinical visit on a visual analogue scale (VAS) consisting of a line ranging from 0 to 100 % [17]. Clear instructions were given as follows; that 0 % meant no medicines had been taken since the last clinic visit, 50 % meant about half the medicines were taken, and 100 % meant that no doses of any of the medicines had been missed. Both questions were used to assess adherence, the first which had been used before in the study population, and the second to capture adherence between visits.

### Acceptability Questionnaires

Carers, and children if appropriate, randomised to PTI were asked to complete an acceptability questionnaire, adapted from a previous PENTA study [16], at baseline (protocol amendment) and again at the end of the study.

Participants were asked to choose from five options about how they thought stopping medicines as part of a planned treatment interruption would affect them and the rest of their family. In addition, at baseline, they were asked if they had any concerns about the possible disadvantages of a PTI, and at the end of the study if they were happy to undergo further PTIs. Other structured questions included asking about problems while on PTIs and with re-starting medicines following a PTI, as well as asking if there was any difference giving or taking medicines before or after a PTI.

### Statistical Analysis

Non-adherence was defined as either reporting one or more missed doses over the last 3 days and/or marking <100 % on the VAS. Due to the different proportion of questionnaires completed in Europe/USA and Thailand, analyses were performed by region as well as by randomised group. As the design of the study meant that the PTI group restarted ART at different times, adherence data from questionnaires from all time points were combined per group and analysed together (including data from questionnaires completed after each ART restart in the PTI group). For comparisons over time, data from each time point were compared (data after the first restart only in the PTI group). Carer or child questionnaires not completed at all at particular time points were omitted from all analyses; individual questions not answered on carer/child questionnaires which had been partly completed were omitted from the analysis of that question. Comparisons of proportions were analysed using Chi-squared (χ^2^) tests and differences in medians using the Wilcoxon rank-sum test. Multilevel univariate logistic regression was used to analyse the results of multiple questionnaires per person completed during follow-up; fixed-effects odds ratios (OR) and confidence intervals (CI) are reported.

## Results

PENTA 11 randomised 110 children from three continents between November 2004 and December 2006: Europe (83 children), USA (4), Thailand (23). One child (randomised in error) was excluded, leaving 109 children (53 CT, 56 PTI) included in the analysis. At baseline the median age was 9 (range 2–16) years, and median CD4 % was 37 % (interquartile range (IQR): 33, 41) (see Table 1 in the main trial publication for further details of the baseline characteristics [14]). In the PTI group, 19 (34 %) children reached the CD4-guided ART restart criteria between 6 and 42 weeks after stopping ART, 32 (57 %) restarted ART because they had been off ART for 48 weeks, four restarted for other reasons and one child did not restart ART for social reasons. Sixteen children had a second PTI, and no child had a third PTI. No child died or had a new CDC C diagnosis, and 1 child (2 %) in the CT group versus 4 (7 %) in the PTI group (difference 5, 95 % CI −2 to +13 %; adjusted OR (95 % CI) = 4.09 (0.42, 39.78), χ^2^ (1) = 1.47, *p* = 0.23) reached a CD4 outcome [14]. However, 50 clinical events, mainly grade 2, were reported in 29 (52 %) PTI children compared with 26 in 15 (28 %) CT children (rate ratio (95 % CI) = 2.35 (1.27–4.39), *z* = 2.87, *p* = 0.004) (see Table 2 in the main trial publication for full details of the clinical events reported [14]).

**Table 1:** Characteristics by randomised group and region

	Europe/USA CT	Europe/USA PTI	Thailand CT	Thailand PTI
Number of children	41 (1 in USA)	45 (3 in USA)	12	11
Age at baseline (years)				
Median (IQR)	10.1 (7.1–12.0)	9.3 (6.8–12.0)	8.0 (5.8–12.9)	8.2 (6.7–11.4)
10 to <16 (%)	21 (54)	19 (42)	5 (42)	3 (27)
Gender (%)				
Male	17 (42)	23 (51)	5 (41)	4 (36)
Ethnic origin (%)				
White	17 (42)	21 (47)	0 (0)	0 (0)
Black	17 (42)	17 (38)	0 (0)	0 (0)
Asian	0 (0)	0 (0)	12 (100)	11 (100)
Other^a^	7 (17)	7 (16)	0 (0)	0 (0)
CD4 %				
Median (IQR)	37 (35–41)	37 (33–43)	35 (32–38)	34 (32–41)
Carer reported non-adherence^b^ at baseline (%)				
Yes	1 (2)	5 (11)	0 (0)	0 (0)
No	25 (61)	20 (44)	12 (100)	11 (100)
Not known	15 (37)	20 (44)	0 (0)	0 (0)
Relative who completed most carer questionnaires^c^ (%)				
Mother	28 (68)	26 (58)	3 (25)	1 (9)
Father	4 (10)	6 (13)	1 (8)	1 (9)
Other carer	1 (2)	7 (16)	8 (67)	9 (82)
Not known	8 (20)	6 (13)	0 (0)	0 (0)
Cumulative ART exposure prior to baseline (years)				
All median (IQR)	7.5 (5.3–9.4)	6.1 (4.6–8.6)	2.9 (1.8–3.9)	2.8 (2.5–3.7)
NRTIs median (IQR)	7.5 (5.3–9.2)	6.1 (4.3–8.3)	2.9 (1.8–3.9)	2.8 (2.5–3.1)
NNRTIs median (IQR)	1.4 (0.0–4.3)	3.2 (0.0–5.1)	2.9 (1.8–3.9)	2.8 (2.5–3.1)
PIs median (IQR)	4.3 (0.0–5.7)	2.7 (0.0–5.3)	0.0	0.0
Child^d^ reported non-adherence^b^ at baseline (%)				
Yes	2 (10)	0 (0)		
No	10 (47)	6 (32)		
Not known	9 (43)	13 (68)		
Knowledge of HIV infection status^c^ (%)				
Yes	16 (39)	15 (33)	8 (67)	7 (64)
No	17 (41)	19 (42)	4 (33)	4 (36)
Not known	8 (20)	11 (24)	0 (0)	0 (0)

*ART* antiretroviral therapy, *CT* continuous therapy, *PTI* planned treatment interruption, *ABC* abacavir, *3TC* lamivudine, *NVP* nevirapine, *EFZ* efavirenz, *d4T* stavudine, *AZT* zidovudine, *IQR* inter-quartile range, *NRTI* nucelos(t)ide reverse transcriptase inhibitor, *NNRTI* non-nucleoside reverse transcriptase inhibitor, *PI* protease inhibitor

^a^Nine children (five Europe/USA CT and four Europe/USA PTI) were South American, four (two Europe/USA CT and two Europe/USA PTI) were mixed black/white and one (Europe/USA PTI) was American Indian

^b^Non-adherence was defined as either reporting one or more missed doses over the last three days or marking <100 % adherence since the last clinical visit on the VAS

^c^On or before the last adherence questionnaire

^d^Only counted as not known if child is >10 years and in Europe/USA. In Thailand questionnaires were only completed by carers

**Table 2:** Questionnaires completed during follow-up by randomised group and region

Total number completed	Carer overall (%)	Carer Europe/USA (%)	Carer Thailand (%)	Children^a^ Europe/USA (%)
Adherence CT^b^				
Overall during follow-up	116/159 (73)	80/123 (65)	36/36 (100)	31/69 (45)
At week 24	38/53 (72)	26/41 (63)	12/12 (100)	7/22 (32)
At week 48	36/53 (68)	24/41 (59)	12/12 (100)	12/23 (52)
At week 72	42/53 (79)	30/41 (73)	12/12 (100)	12/24 (50)
Adherence PTI^c^				
Overall during follow-up	91/205 (44)	54/166 (33)	37/39 (95)	35/76 (46)
After first re-start^d^	84/188 (45)	50/152 (33)	34/36 (94)	27/62 (44)
At week 4	20/53 (38)	12/44 (27)	8/9 (89)	7/19 (37)
At week 12	22/53 (42)	14/44 (32)	8/9 (89)	7/19 (37)
At week 24	26/50 (52)	17/41 (42)	9/9 (100)	9/17 (53)
At week 48	16/32 (50)	7/23 (30)	9/9 (100)	4/7 (57)
After second re-start	7/17 (41)	4/14 (29)	3/3 (100)	8/14 (57)
At week 4	3/7 (43)	2/6 (33)	1/1 (100)	2/5 (40)
At week 12	2/6 (33)	1/5 (20)	1/1 (100)	2/5 (40)
At week 24	2/4 (50)	1/3 (33)	1/1 (100)	3/3 (100)
At week 48				1/1 (100)
Acceptability PTI				
Baseline^e^	18/35 (51)	15/26 (58)	3/9 (33)	9/15 (60)
End of the study	37/56 (66)	27/45 (60)	10/11 (91)	14/23 (61)
Children with at least one				
Adherence CT	49/53 (92)	37/41 (90)	12/12 (100)	21/26 (81)
Adherence PTI	46/56 (82)	35/45 (78)	11/11 (10)	22/26 (85)
Acceptability PTI	43/56 (77)	33/45 (73)	10/11 (91)	19/25 (76)

*CT* continuous therapy, *PTI* planned treatment interruption

^a^Only counted as missing if child is >10 years

^b^Carer: OR (week 48 vs. 24, 95 % CI) = 0.78 (0.29, 2.08), OR (week 72 vs. 24, 95 % CI) = 1.75 (0.61, 5.02), χ^2^ (2) = 2.35, *p* = 0.31

Children: OR (week 48 vs. 24, 95 % CI) = 3.92 (0.74, 20.82), OR (week 72 vs. 24, 95 % CI) = 3.31 (0.64, 17.14), χ^2^ (2) = 2.93, *p* = 0.23

^c^Two children restarted ART at the end of the study, one child did not restart ART

^d^Carer: OR (week 12 vs. 4, 95 % CI) = 1.42 (0.44, 4.57), OR (week 24 vs. 4, 95 % CI) = 3.43 (0.99, 11.80), OR (week 48 vs. 4, 95 % CI) = 1.65 (0.42, 6.46), χ^2^ (3) = 3.99, *p* = 0.26

Children: OR (week 12 vs. 4, 95 % CI) = 1.00 (0.16, 6.33), OR (week 24 vs. 4, 95 % CI) = 3.51 (0.50, 24.59), OR (week 48 vs. 4, 95 % CI) = 3.54 (0.27, 47.05), χ^2^ (3) = 2.44, *p* = 0.49

^e^Only counted as missing if after the protocol amendment: October 2005

At baseline, carer reported non-adherence was similar across randomised groups, but carers in Europe/USA reported non-adherence more often than in Thailand (Table 1; Europe/USA 6/51, 12 %; Thailand 0/23, 0%; χ^2^ (1) = 2.94, *p* = 0.09). Carer questionnaires in Europe/USA were mainly completed by the mother/father (mother: 54/86, 63 %; father: 10/86, 12 %), while in Thailand it was predominantly another carer (17/23, 74%; χ^2^ (2) = 35.97, *p* < 0.001). Additionally children enrolled in Europe/USA had been exposed to ART for longer than those from Thailand (median [IQR]: Europe/USA 7.0 years [4.7, 9.2], Thailand 2.9 years [2.0, 3.9]; *z* = −5.49, *p* < 0.001), but fewer were reported to have knowledge of their infection status (Europe/USA 31/67, 46 %; Thailand 15/23, 65 %). Children in Europe/USA reported similar baseline non-adherence across randomised groups (Table 1); children in Thailand did not complete questionnaires.

### Adherence Questionnaires During Follow-Up

At least one adherence questionnaire was completed during follow-up by a carer for 49/53 (92 %) children in the CT and 46/56 (82 %) in the PTI group (χ^2^ (1) = 2.59, *p* = 0.11) (Table 2). The proportion of returned carer questionnaires was higher in Thailand (73/75, 97 %) compared to Europe/USA (134/289, 46 %;OR (95 % CI) = 0.0072 (0.00097, 0.054), χ^2^ (1) = 23.12, *p* < 0.001). The number of questionnaires completed did not differ over time in either randomised group for carers or children (Table 2).

The question on missed doses over the last 3 days was answered on 182/207 (88 %) carer questionnaires and the VAS was marked on 160/207 (77 %). 2/194 (1 %) reported both missed doses and marked at <100 % on the VAS, 5 (3 %) reported missed doses but did not mark the VAS, and 25 (13 %) marked at <100 % on the VAS (of which 19 reported no missed doses in the last 3 days and 6 did not answer the question). This led to an overall non-adherence rate of 32/194 (16 %) during follow-up.

Figure 1 shows the reported non-adherence by randomised group and region. There was no difference in carer reported non-adherence between CT and PTI groups (18 vs. 14 % respectively; OR (95 % CI) = 1.04 (0.20, 5.41), χ^2^ (1) = 0.003, *p* = 0.96) and it did not differ over time on CT (week 24: 14 % (5/37), week 48: 20 % (7/35), week 72: 21 % (8/39); χ^2^ (2) = 1.56, *p* = 0.46) or after first re-start in PTI group (week 4: 21 % (4/19), week 12: 11 % (2/19), week 24: 21 % (5/24), week 48: 0 % (0/14); χ^2^ (2) = 1.00, *p* = 0.61, week 48 omitted from model). Carers reported non-adherence more often in Europe/USA than in Thailand (26 vs. 1 % respectively; OR (95 % CI) = 54.65 (3.68, 810.55), χ^2^ (1) = 8.45, *p* = 0.004).

Children from Europe/USA who completed questionnaires were of a median (IQR) age of 11 (10–14) years. Non-adherence was reported by these children on 35 % (22/63) of questionnaires but did not differ by randomised groups (Fig. 1, CT 30 %; PTI 39 %; OR (95 % CI) = 3.10 (0.25, 37.79), χ^2^ (1) = 0.79, *p* = 0.38) or over time (CT χ^2^ (2) = 1.09, *p* = 0.58; PTI after first re-start, χ^2^ (3) = 4.20, *p* = 0.24). On the 36 occasions when both the child and their carer completed separate questionnaires, non-adherence was reported by both on eight occasions and only the child on 2.

### Acceptability Questionnaires

Small numbers of acceptability questionnaires were completed at baseline as these were only included in a protocol amendment after the trial had started (October 2005). Overall questionnaire return rates among carers who were offered questionnaires were 13/20 (65 %) in Thailand and 42/71 (59 %) in Europe/USA (OR (95 % CI = 0.79 (0.23, 2.73), χ^2^ (1) = 0.14, *p* = 0.71) (Table 2).

**Fig. 2:**
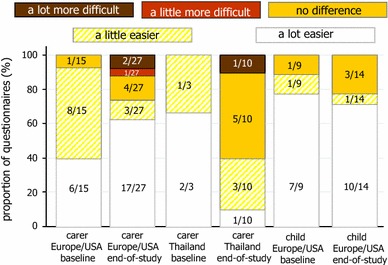
*PTI* planned treatment interruption. Carer and child responses to the question “How do you think (baseline)/did (end of study) stopping medicines as part of a PTI make things for you?”, by randomised group and region

**Fig. 3:**
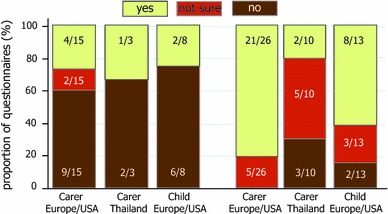
*PTI* planned treatment interruption. Carer and child responses to the questions “Do you have any concerns about the possible disadvantages of a PTI?” (baseline, left graph) and “Are you happy to have further PTIs?” (end of study, right graph), by randomised group and region

At baseline, 94 % (17/18) of carers and 89 % (8/9) of children thought PTIs would make life easier, decreasing to 65 % (24/37, χ^2^ (1) = 2.02, *p* = 0.16) of carers and 79 % (11/14, χ^2^ (1) = 0.37, *p* = 0.54) of children by the end of the study; Europe/USA carers reported PTIs more favourably (overall: Europe/USA easier 34/42, 81 %; Thailand 7/13, 54 %; χ^2^ (1) = 3.84, *p* = 0.05) (Fig. 2). At baseline only 5/18 (28 %) of carers (Europe/USA 4/15, 27 %; Thailand 1/3, 33 %) and 2/8 (25 %) children had concerns about the possible disadvantages of PTI, and at the end of the study most carers (23/36, 64 %) and children (8/13, 62 %) were happy to have further PTIs (Fig. 3). However, again there were differences by region; in Europe/USA 21/26 (81 %) carers said they were happy, but in Thailand opinion was split (yes 2/10, 20 %; no 3/10, 30 %; not sure 5/10, 50 %; χ^2^ (1) = 11.56, *p* = 0.001).

Approximately half of the carers (Europe/USA 12/26, 46 %; Thailand 3/10, 30 %) and children (6/12, 50 %) at the end-of study had found more clinic visits during PTI a problem. Two thirds of children said they did not want to start medicines again following a PTI (7/11, 64 %), but a lower proportion of carers reported this being a problem (Europe/USA 7/20, 35 %, Thailand 2/10, 20 %). When asked if there was any difference giving/taking medicines before and following a PTI, most carers (Europe/USA 16/25, 64 %; Thailand 8/10, 80 %) and children (9/13, 69 %) said there was no difference.

Few carers and children said feeling ill (carer: Europe/USA 3/22, 14 %; Thailand 2/10, 20 %; children: 1/12, 8 %), developing minor signs/symptoms (carer: Europe/USA 6/22, 27 %; Thailand 0/10, 0 %; children: 0/12, 0 %), or feeling anxious (carer: Europe/USA 4/17, 24 %; Thailand 1/10, 10 %; children: 1/11, 9 %) during a PTI were problems. Of the 29 children in the PTI group with reported clinical events, few at the end of the study reported feeling ill (1/16), developing minor signs/symptoms (3/15) or feeling anxious (4/15) were problems.

## Discussion

Overall, reported adherence to ART was similar in the CT and PTI groups, and participants reported that PTIs generally made life easier. The majority of children and carers were happy to have further PTIs, and only a few experienced problems with treatment interruptions.

PTIs did not seem to impact negatively on adherence, and the reported adherence rate remained stable over time. These observations are similar to those reported in the Trivacan ANRS 1269 trial in adults [9], where non-adherence rates, measured by the patient reporting ≥1 missed dose in the last 4 days, were 11.1 % in the CT group and 11.3 % in the interruption group. Reported adherence was also comparable to previous non-interruption studies in children, which used similar questionnaires. For example, PACTG 219C [18] reported “missed doses over the 3 days before the study visit” for 324/2088 (16 %) subjects and PENTA 5 [15] reported “forgot one or more doses in the last 7 days” on 69/265 (26 %) questionnaires. A recent literature review on paediatric adherence to ART [19] revealed a comparable overall adherence rate of 73 % when adherence was assessed by caregiver report.

Better adherence was reported in Thailand than Europe/USA. This is in line with the recent literature review [19], where adherence was better in low/middle income compared to high-income countries. Correspondingly, Thai carers found PTIs less acceptable, and it therefore appears carers in Thailand preferred to give ART medication routinely. There could be a number of reasons for this observation. Firstly, Thai carers may have had concerns about their children falling ill when ART was stopped; ART only became widely available in Thailand in the last few years and before ART was introduced, about 500,000 adults and 12,000 children died of AIDS [20]. Secondly, most questionnaires in Thailand were completed by a carer other than the child’s parents. It is possible that the non-biological parents in Thailand, usually grandparents or relatives, have witnessed one or more family members dying of AIDS; therefore, they are more concerned about the children not being on continuous ART. Lastly, at baseline, all children from Thailand had only been exposed to two ART classes and, on average, had taken ART for a much shorter period of time (median <3 years) than children in Europe/USA. Therefore the desire to stop medication may have been less than is Europe/USA, where children had already taken ART for a median of 7 years.

An excess of minor clinical events; mainly haematological/lymphatic system disorders, dermatological (rashes) and CNS/psychiatric (most commonly headaches), were observed in PTI group of PENTA 11 [14]. However, these events did not appear to impact on acceptability of PTIs. Instead, carers and children reported that practical issues, such as more clinic visits and re-starting medication, were more problematic than illness. Though mainly descriptive, comments on acceptability questionnaires were of interest. They were mainly positive, and included “*She will have a break, she can enjoy staying over with friends and family who don*’*t know her diagnosis*”, “*no need to wake up early*”, “*I have been very well without treatment*”, and “*The child had normal life*”. However, a number of children commented on practical problems; “*I have now restarted the medication and I am finding this more difficult because of short term side effects*”, and “*I had to go to the clinic every* 2 *weeks*”.

Although using a different tool in smaller numbers, our results differ from the SMART [13] and DART [21] trials in adults. In SMART a total of 1225 patients had QOL assessments over a mean follow-up time of 2.4 years, and whenever QOL outcomes differed, the results were inferior among patients in the CD4-guided episodic therapy group compared with the CT group; excluding participants with disease progression had minimal effect on QOL comparisons [13]. Participants in the DART trial completed acceptability questionnaires at termination of structured treatment interruption (STI) (*n* = 408): 36 % reported STIs made things “*a little/lot harder*”, 31 % “*no different*” and 32 % “*a little/lot easier*” [21]. Similarly, higher proportions of participants in the STI group within DART reported feeling ill (38 %) or anxious (42 %) was a problem during STI, than in PENTA 11. However comparable proportions of DART participants said they were willing to interrupt ART again (62 %). Of note, CD4 counts at interruption in both SMART and DART trials were lower than in PENTA 11; however, although DART patients had late stage HIV disease, it is also true that 26 % of children in PENTA 11 had experienced an AIDS event prior to entry.

Two smaller studies in adults observed no difference in QOL for patients experiencing STIs. Firstly, the STACCATO trial (*n* = 379) [22] observed no difference in QOL or mental health between the CT and CD4-guided interruption arms. Secondly, the AIDS Clinical Trials Group 5170 observational study of 167 asymptomatic HIV-infected patients who wished to discontinue ART found QOL did not change during a prolonged treatment interruption [23].

There are several limitations of this study; firstly, the low questionnaire return rate, particularly in the PTI group within Europe/USA. Although reasons for not completing a questionnaire were not formally collected, comments from staff at the participating European sites were that they were reluctant to give patients the questionnaires so regularly, as families already had lengthy clinic appointments, especially in the PTI group. Secondly, the small sample size of children from Thailand means the role of bias when comparing Europe/USA and Thailand cannot be ruled out. Thirdly, the assessment of adherence was through self-reported measures and therefore the results presented are a subjective measure of adherence. Also, the VAS had not been used to assess adherence in this population previously. Lastly, combining adherence data from questionnaires from all time points per group meant that comparisons between groups at specific time points were not made.

PENTA 11 was a pilot study in children, and the strategy of treatment interruptions needs further evaluation within a larger trial to clarify whether PTIs have a future role in paediatric HIV management. Two large African paediatric treatment interruption trials will report shortly (BANA II [24] and CHER [25]), and will further assess the impact of PTIs on the QOL in children. However, in the subset of children who answered questionnaires, treatment interruptions do not appear to have a negative impact on reported adherence, although adherence was lower in Europe/USA than Thailand. The acceptability of PTIs was reasonable in this small sample, with the majority of families indicating they were happy to have further PTIs. Increased clinic visits and re-starting ART after PTI appeared to be the main issues with treatment interruption.

## Appendix: Paediatric European Network for Treatment of AIDS (PENTA) 11 Trial Team

*PENTA Steering Committee*: J-P Aboulker, J Ananworanich, A Babiker, E Belfrage, S Bernardi, S Blanche, A-B Bohlin, R Bologna, DM Burger, K Butler, G Castelli-Gattinara, H Castro (nee Green), P Clayden, A Compagnucci, JH Darbyshire, M Debré, A Faye, R de Groot, M della Negra, D Duiculescu, C Giaquinto (chairperson), DM Gibb, I Grosch-Wörner, M Hainault, L Harper, N Klein, M Lallemant, J Levy, H Lyall, M Marczynska, M Mardarescu, MJ Mellado Peña, D Nadal, T Niehues, C Peckham, D Pillay, JT Ramos Amador, L Rosado, R Rosso (deceased), C Rudin, Y Saidi, HJ Scherpbier, M Sharland, M Stevanovic, C Thorne, PA Tovo, G Tudor-Williams, N Valerius, AS Walker, S Welch, U Wintergerst.

*PENTA 11 Executive Committee*: J-P Aboulker, A Babiker, DM Burger, A Compagnucci, JH Darbyshire, M Debré, C Giaquinto, DM Gibb, H Castro (nee Green), L Harper, N Klein, M Lallemant, H Lyall, L Mofenson, J Moye, D Nadal, Y Saïdi.

*PENTA 11 Pharmacology Group*: DM Burger, TR Cressey, E Jacqz-Aigrain, S Khoo, JM Tréluyer.

*PENTA 11 Immunology/Virology Group*: A De Rossi, N Klein, J Moye, N Ngo-Giang-Huong, MA Muñoz Fernandez, D Pillay.

*PENTA 11 Data Safety and Monitoring Committee*: C Hill (chairperson), P Lepage, A Pozniak, S Vella.

*Trials centres*:

*INSERM SC10*, *France*: J-P Aboulker, A Compagnucci, G Hadjou, S Léonardo, Y Riault, Y Saïdi.

*MRC Clinical Trials Unit*, *UK*: A Babiker, L Buck, JH Darbyshire, L Farrelly, S Forcat, DM Gibb, H Castro (nee Green), L Harper, L Harrison, J Horton, D Johnson, S Moore, C Taylor, AS Walker.

*PHPT*, *Thailand*: S. Chalermpantmetagul, TR Cressey, R. Peongjakta, S. Chailert, F Fregonese, G Jourdain, M Lallemant, N Ngo-Giang-Huong.

*Westat*/*NICHD*, *USA*: D Butler, C Carlton, D Collins, G Kao, L Mofenson, J Moye, S Van Buskirk, S Watson.

Clinical sites (Pharmacists (P), Virologists/immunologists(L)):

*France*: Hôpital Femme-Mère-Enfant, Lyon: S Corradini, D Floret, TT Le Thi (L); Hôpital de l’Archet, Nice: F Monpoux, J Cottalorda, JC Lefebvre, S Mellul; Hôpital Cochin Port-Royal, Paris: N Boudjoudi, G Firtion, M Denon, F Picard (L); Hôpital Robert Debré, Paris: A Faye, D Beniken (L), F Damond (L); G.Alexandre (L); Hôpital Purpan, Toulouse: J Tricoire, F Nicot (L); Hôpital Saint-Vincent de Paul, Paris: A Krivine (L); D Rivaux; Hôpital Necker, Paris: ML Chaix.

*Germany*: Universitäts-kinderkliniken, Munich: U Wintergerst, G Notheis, G Strotmann, S Schlieben.

*Italy*: Università di Padova: C Giaquinto, O Rampon, M Zanchetta; Università di Genova: R Rosso, F Ginocchio, C Viscoli; Ospedale Bambino Gesù, Rome: G Castelli-Gattinara, S Bernardi, A Martino, G Pontrelli, C Concato (L); Ospedale S. Chiara, Trento: A Mazza, G Rossetti (L).

*Poland*: Medical University of Warsaw/Regional Hospital of Infectious Diseases, Warsaw: M Marczynska, S Dobosz, A Oldakowska, J Popielska, M Doroba, J Stanczak, G Stanczack, T Dyda.

*Spain*: Hospital Universitario 12 de Octubre, Madrid: MI González Tomé, R Delgado García, MT Fernandez Gonzalez; Hospital Carlos III, Madrid: M José Mellado Peña, P Martín-Fontelos, R Piñeiro Pérez, M. Penin, I Garcia Mellado, AF Medina, B Ascencion (L); Hospital Universitario de Getafe, Madrid: JT Ramos Amador, I Garcia Bermejo (L), JA Garcia Vela (L), I Martin Rubio (L); Hospital General Universitario Gregorio Marañón, Madrid: D Gurbindo, ML Navarro Gomez, JL Jimenez (L), MA Muñoz-Fernandez (L), A. Garcia Torre (L); Hospital Infantíl La Paz, Madrid: MI de José Gómez, MC García Rodriguez; Hospital Materno-Infantil, Málaga: D Moreno Pérez, E Núñez Cuadros; Hospital Infantil La Fe, Valencia: F Asensi-Botet, A Pérez, MD Pérez Tamarit, M Gobernado Serrano (L), A Gonzales Molina (L).

*Switzerland*: University Chidren’s Hospital, Zurich: C Kalhert, D Nadal, M Dobrovoljac, C Berger, G Nobile, S Reinhard (L); National Laboratory for Retrovirus, Zurich: J.Schupbach.

*Thailand*: HIV-NAT, the Thai Red Cross AIDS Research Centre, Bangkok: T Bunupuradah, J Ananworanich, T Puthanakit, C Pancharoen, O Butterworth, C Phasomsap, T Jupimai, S Ubolyam (L), P Phanuphak; Nakornping Hospital, Chiang Mai: S Kanjanavanit, T Namwong, D Chutima (P) and M Raksasang (L).

*UK*: Imperial College Hospital Healthcare Trust, London: H Lyall, G Tudor-Williams, C Foster, D Hamadache, S Campbell, C Newbould, C Monrose, D Patel (P), S Kaye (L); Chelsea and Westminster Hospital, London: H Lyall, P Seery, D Hamadache, A Wildfire (L); Great Ormond Street Hospital for Children, London: V Novelli, DM Gibb, N Klein, D Shingadia, K Moshal, J Flynn, M Clapson, L Farrelly, A Allen, L Spencer, M Depala (P), M Jacobsen (L); University Hospital of North Staffordshire, Stoke on Trent: P McMaster, M Phipps, J Orendi, C Farmer; Newham University Hospital, London: S Liebeschuetz, O Sodeinde, D Shingadia, S Wong; Birmingham Heartlands Hospital: S Welch, Y Heath, S Scott, K Gandhi (P); University Hospitals Coventry & Warwickshire NHS Trust University Hospital: P Lewis, J Daglish; Royal Free and University College Medical School, London: D Pillay (L).

*USA*: SUNY Upstate Medical University, Syracuse NY: L Weiner, M Famiglietti**;** Howard University Hospital, Washington DC: S Rana, P Yu, J Roa; Children’s Diagnostic & Treatment Center, Ft. Lauderdale, FL: A Puga, A Haerry, A Inman.
